# Exceptional association of two species of bacteria causing acute appendicitis: Haemophilus influenzae and Enterobacter cloacae

**DOI:** 10.1099/acmi.0.000794.v3

**Published:** 2024-05-28

**Authors:** Fatima Zahra Adil, Elmostafa Benaissa, Yassine Ben Lahlou, Leila Laamara, Fatna Bssaibis, Adil Maleb, Mariama Chadli, Mostafa Elouennass

**Affiliations:** 1 Bacteriology Department, Mohammed V Military Teaching Hospital, Rabat, Morocco; 2 Research Team of Epidemiology and Bacterial Resistance, Faculty of Medicine and Pharmacy, Mohammed V University, Rabat, Morocco

**Keywords:** acute appendicitis, children, *Enterobacter cloacae*, *Haemophilus influenzae*

## Abstract

Appendicitis, typically caused by appendiceal lumen obstruction, is a prevalent abdominal surgical emergency worldwide. While most cases involve *Enterobacterales, Haemophilus influenzae*, primarily known for upper respiratory infections, is infrequently associated with gastrointestinal infections. This article presents an exceptional case of acute appendicitis caused by both *Haemophilus influenza* and *Enterobacter cloacae* in a 15-year-old child, highlighting the significance of recognizing uncommon pathogens in appendicitis and emphasizing the necessity for thorough microbiological investigations to refine diagnostic approaches.

## Data Summary

No data was generated during this research or is required for the work to be reproduced.

## Introduction

Appendicitis refers to the inflammation of the vermiform appendix; its primary cause is typically attributed to the obstruction of the appendiceal lumen [1]. Acute appendicitis stands as the most prevalent abdominal surgical emergency worldwide [2]. Most often, the bacteria associated with acute appendicitis include species of *Enterobacterales* like *Escherichia coli*, alongside other genera such as *Bacteroides, Peptostreptococcus*, and *Pseudomonas* [1].

*Haemophilus influenzae* is a part of the normal microbiota of the upper respiratory tract. It is responsible for various infections ranging from uncomplicated upper respiratory conditions such as conjunctivitis, sinusitis, and otitis media to severe ones such as endocarditis and meningitis [3]. However, little is known about the ability of *Haemophilus influenzae* to cause gastrointestinal infections [4].

We report an exceptional case of acute appendicitis due to both *Haemophilus influenzae* and *Enterobacter cloacae* in a 15-year-old child.

## Case presentation

A 15-year-old child was admitted to the emergency department of the Mohamed V Military Teaching Hospital (MVMTH) for intense pain in the right iliac fossa, where abdominal guarding was observed during the clinical examination. Notably, the patient was afebrile during the evaluation. Among the patient’s medical history is a tonsillectomy performed at the age of eight.

The biological assessment revealed a white blood cell count of 13 100 µl^−1^ and a C-reactive protein of 16 mg l^−1^. Abdominal ultrasound showed a swollen aperistaltic and non-compressible appendix with an outer diameter measuring 8 mm associated with hyperechoic periappendiceal fat stranding and reactive lymphadenopathy.

The diagnosis made was acute non-perforated appendicitis. The patient subsequently underwent an appendectomy using the McBurney incision. Swabbing of the appendiceal base was performed, and the sample was sent to the microbiology laboratory of MVMTH.

**Fig. 1.**
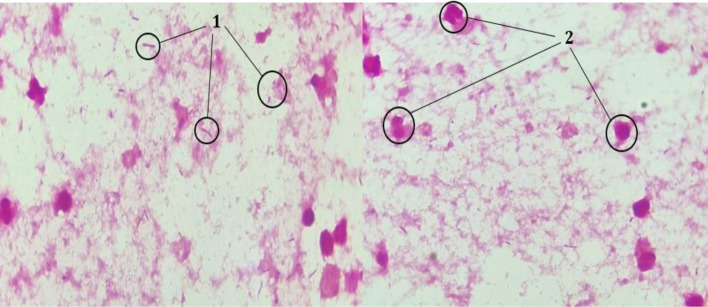
Gram-negative bacilli (1) and neutrophilic polymorphonuclear cells (2) on a Gram-stained smear.

The sample was inoculated on both blood agar and enriched chocolate agar, then incubated at 37 °C in aerobic conditions with CO_2_. Additionally, Schaedler agar and blood agar supplemented with colistin and nalidixic acid were employed and incubated under anaerobic conditions. A smear for Gram-staining was performed, revealing a cellular reaction composed of neutrophilic polymorphonuclear cells and numerous Gram-negative bacilli (Fig. 1).

**Fig. 2.**
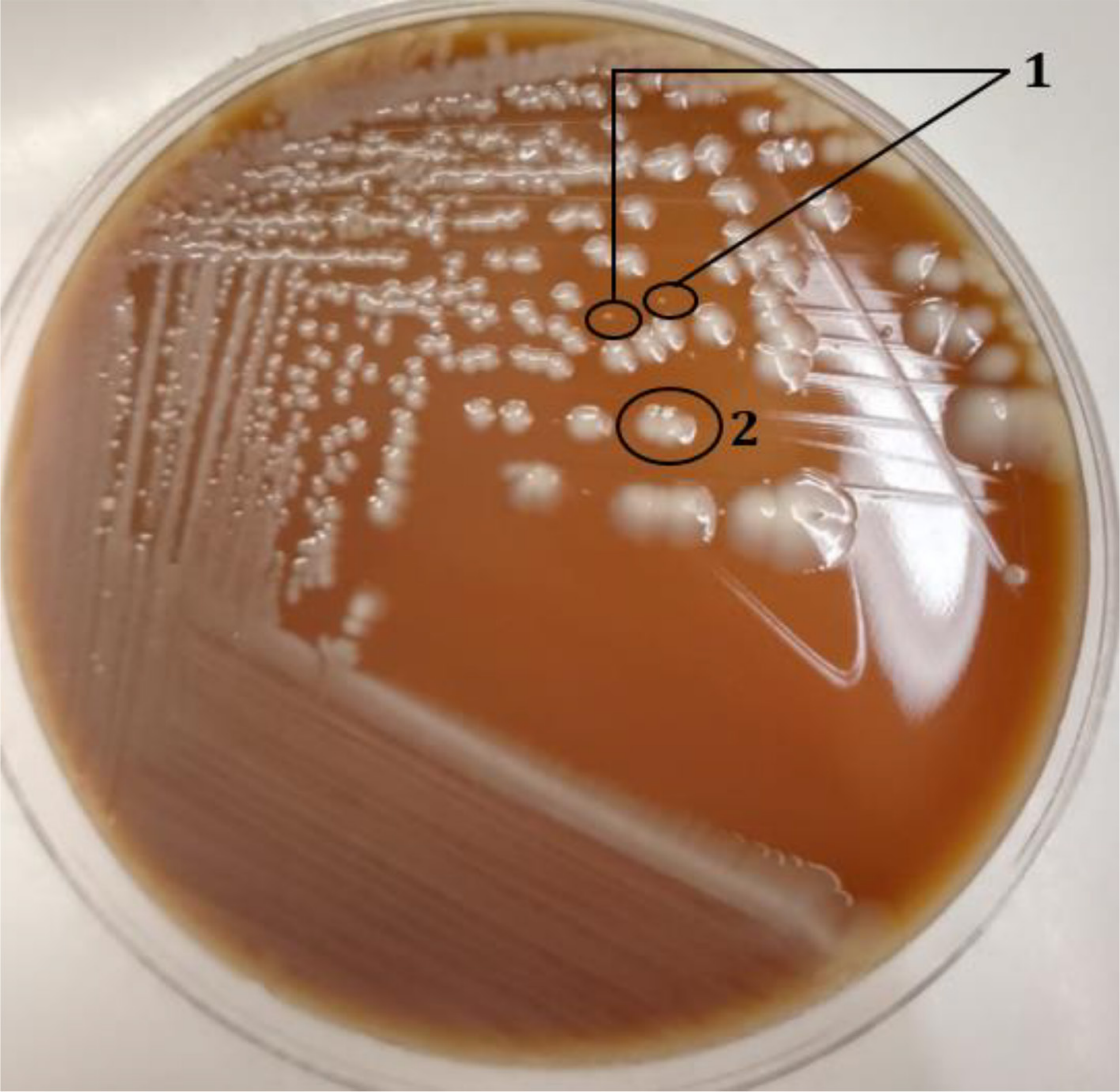
Colonies of *Haemophilus influenzae* (1) and *Enterobacter cloacae* (2) on enriched chocolate agar.

Following a 24 h incubation period, the aerobic culture yielded positive results, revealing two distinct types of Gram-negative bacilli colonies on chocolate agar: one characterized by large colonies and the other by smaller ones (Fig. 2). Conversely, only the large colonies were evident on blood agar. In regard to anaerobic culture, after 48 h of incubation, the large colonies were detected on Schaedler agar, while no growth was noted on blood agar supplemented with colistin and nalidixic acid. No colonies indicative of anaerobic bacteria were observed.

Species identification was based on morphological and biochemical characteristics using API gallery, bio-Mérieux SA, Marcy l’Étoile, France. The results indicated *Haemophilus influenzae* and *Enterobacter cloacae* with probabilities of 99.9 % and 98.7 %, respectively. Antimicrobial susceptibility testing was performed using the disc diffusion technique following the recommendations of the Antibiotic Susceptibility Committee of the French Society of Microbiology (CA-SFM) and the European Committee on Antimicrobial Susceptibility Testing (EUCAST) [5]. Antibiograms were conducted using 0.5 McFarland suspensions on chocolate agar for *Haemophilus influenzae* and Müller-Hinton agar for *Enterobacter cloacae*. They were subsequently incubated at 37 °C under aerobic conditions. CO_2_ supplementation was added specifically for the antibiogram of *Haemophilus influenzae*. The results demonstrated that both *Haemophilus influenzae* and *Enterobacter cloacae* were susceptible (Table 1).

**Table 1.** Results of antimicrobial susceptibility testing (PG: Penicillin G, AMP: Ampicillin, TIC: Ticarcillin, AMC: Amoxicillin-Clavulanic Acid, TZP: Piperacillin-Tazobactam, CFR: Cefadroxil, FOX: Cefoxitin, CTX: Cefotaxime, CFM: Cefixime, FEP: Cefepime, ERT: Ertapenem, IMP: Imipenem, NA: Nalidixic acid, CIP: Ciprofloxacin, LVX: Levofloxacin, TET: Tetracycline, MIN: Minocycline, RIF: Rifampicin, SXT: Trimethoprim-Sulfamethoxazole, AK: Amikacin, GN: Gentamicin, TOB: Tobramycin)

*Haemophilusinfluenzae*	PG	AMP	TZP	CTX	CFM	FEP	ERT	IMP	NA	CIP	LVX	TET	MIN	RIF	SXT	
	S	S	S	S	S	S	S	S	S	S	S	S	S	S	S	
*Enterobactercloacae*	AMP	TIC	AMC	TZP	CFR	FOX	CTX	FEP	IMP	AK	GN	TOB	CIP	LVX	FOS	SXT
	R	S	R	S	R	R	S	S	S	S	S	S	S	S	S	S

The patient was hospitalized for 48 h postoperatively and was prescribed Amoxicillin-Clavulanic Acid 1 g/8 h. A positive clinical progress was observed, and the patient was discharged from the hospital.

## Discussion

In 2019, the global prevalence of acute appendicitis was estimated at approximately 17.7 million cases, equating to an incidence rate of 228 cases per 100 000 population. The associated mortality reached a rate of 0.43 deaths per 100 000 population. The highest incidence occurred in the age range of 15 to 19 [6]. Acute appendicitis has a male-to-female ratio of 1.4 [7].

The most common trigger for appendicitis is typically an obstruction within the appendiceal lumen, often caused by an appendicolith or other mechanical factors like appendiceal tumours. When the appendiceal lumen gets obstructed, bacteria accumulate, leading to acute inflammation and, in some cases, perforation and the formation of abscesses. In the initial stages of appendicitis, aerobic organisms tend to dominate, while as the condition progresses, a combination of both aerobes and anaerobes becomes prevalent [1]. Acute appendicitis is a polymicrobial infection [8]. The most frequently isolated bacteria in many reported cases are Gram-negative bacilli, specifically *Escherichia coli*, *Bacteroides fragilis* and *Pseudomonas aeruginosa*. Additionally, Gram-positive bacteria such as *Streptococcus* spp. and *Clostridium perfringes* have also been found in the appendix [8,9].

*Enterobacter cloacae*, a member of the *Enterobacterales* family residing within the digestive tract [3], has been identified as one of the potential causative agents of appendicitis, as concluded in the study conducted by Yu *et al*. [10].

*Haemophilus influenzae* is a small Gram-negative bacillus [3], commonly associated with respiratory infections, but infrequently responsible for infections in other anatomical sites [11].

The involvement of *Haemophilus influenzae* in appendicitis has been documented in the literature. In 1991, Astagneau *et al*. reported a case of appendicitis involving both *Haemophilus influenzae* and *Streptococcus pneumoniae* in a 4-year-old child [12]. Furthermore, in 1996, another noteworthy case involving an appendiceal mass attributed to *Haemophilus influenzae* was documented in a 3-year-old child [11]. Shedding light on the prevalence of *Haemophilus* spp. in appendicitis cases among children, Mégraud *et al*.’s study revealed the isolation of *Haemophilus* spp. in 7.8 % of operative specimens [13].

The pathophysiological mechanism underlying *Haemophilus influenzae*-induced appendicitis remains elusive. Nevertheless, several hypotheses have been proposed to elucidate its migration to the gastrointestinal tract. One plausible scenario involves a hematogenous route, particularly following prior respiratory tract surgery [12], or an alternative pathway through the descent from the oropharyngeal sphere to the digestive system [4].

## Conclusion

This instance of *Haemophilus influenzae* and *Enterobacter cloacae* co-occurring in acute appendicitis underscores the importance of considering unconventional pathogens in appendicular pathology. Despite unclear pathophysiological mechanisms, the case underscores the need for heightened clinical awareness and comprehensive microbiological investigations. These findings contribute to refining diagnostic approaches and highlight the evolving spectrum of infectious etiologies in appendicitis.

## Abbreviations

AK: amikacin
AMC: amoxicillin-clavulanic acid
AMP: ampicillin
CA-SFM: antibiotic susceptibility committee of the French society of microbiology
CFM: cefixime
CFR: ceradroxil
CIP: ciprofloxacin
CTX: cefotaxime
ERT: ertapenem
EUCAST: European Committee on Antimicrobial Susceptibility Testing
FEP: cefepime
FOX: cefoxitin
GN: gentamicin
IMP: imipenem
LVX: levofloxacin
MIN: minocycline
MVMTH: Mohamed V Military Teaching Hospital
NA: nalidixic acid
PG: penicillin g
RIF: rifampicin
SXT: trimethoprim-sulfamethoxazole
TET: tetracycline
TIC: ticarcillin
TOB: tobramycin
TZP: piperacillin-tazobactam
